# Supplementary material for: Impact of temperature on patient-derived dengue virus breakthrough infections in wMel-infected Aedes aegypti
Source: PLoS Negl Trop Dis. 2026 Oct 9;20(10):e0014255. doi: 10.1371/journal.pntd.0014255 (PMC13686452; doi:10.1371/journal.pntd.0014255)
Supplement: Supplementary file 1 [file pntd.0014255.s001.docx]

**Supp. Table 1. DENV infection (head/thorax positive) and transmission (saliva positive) in WT and *w*Mel-*Ae. aegypti* reared under standard or elevated temperatures.**

| **Serotype** | **Patient RNA-emia titre** | **Head/thorax DENV positive (*n*)** | | | | **Saliva DENV positive (*n*)** | | | |
| --- | --- | --- | --- | --- | --- | --- | --- | --- | --- |
|  |  | **WT** | | ***w*Mel** | | **WT** | | ***w*Mel** | |
|  |  | 28◦C (± 4°C) | 31◦C (± 4°C) | 28◦C (± 4°C) | 31◦C (± 4°C) | 28◦C (± 4°C) | 31◦C (± 4°C) | 28◦C (± 4°C) | 31◦C (± 4°C) |
| DENV-1 | 4.19 x 10^8^ | 20 (20) | 20 (20) | 11 (12) | 20 (20) | 18 (20) | 18 (20) | 5 (12) | 16 (20) |
| DENV-1 | 4.91 x 10^8^ | 20 (20) | 20 (20) | 19 (20) | 15 (16) | 12 (20) | 18 (20) | 12 (20) | 13 (16) |
| DENV-1 | 9.88 x 10^8^ | 9 (9) | 20 (20) | 11 (18) | 10 (11) | 9 (9) | 18 (20) | 4 (18) | 9 (11) |
| **DENV-1 combined %** | | **100** | **100** | **82** | **96** | **80** | **90** | **42** | **81** |
| DENV-2 | 2.03 x 10^5^ | 20 (20) | 19 (19) | 1 (20) | 19 (20) | 11 (20) | 15 (19) | 1 (20) | 9 (20) |
| DENV-2 | 1.58 x 10^6^ | 10 (14) | 12 (17) | 0 (2) | 6 (7) | 7 (14) | 9 (17) | 0 (2) | 6 (7) |
| DENV-2 | 8.57 x 10^6^ | 3 (3) | 6 (6) | 1 (3) | 9 (13) | 2 (3) | 4 (6) | 0 (3) | 3 (13) |
| DENV-2 | 1.03 x 10^7^ | 20 (20) | 0 (1) | 1 (20) | 15 (20) | 8 (20) | 0 (1) | 0 (20) | 10 (20) |
| DENV-2 | 8.51 x 10^7^ | 20 (20) | 18 (18) | 8 (20) | 11 (11) | 9 (20) | 16 (18) | 4 (20) | 8 (11) |
| DENV-2 | 9.70 x 10^7^ | 13 (13) | 18 (19) | 6 (6) | 19 (20) | 1 (13) | 3 (19) | 1 (6) | 4 (20) |
| DENV-2 | 1.06 x 10^8^ | 10 (10) | 17 (17) | 9 (9) | 7 (7) | 5 (10) | 6 (17) | 5 (9) | 6 (7) |
| DENV-2 | 2.22 x 10^8^ | 20 (20) | 16 (16) | 8 (17) | 15 (15) | 12 (20) | 12 (16) | 4 (17) | 12 (15) |
| **DENV-2 combined %** | | **97** | **94** | **35** | **89** | **46** | **58** | **15** | **51** |
| DENV-4 | 5.69 x 10^7^ | 20 (20) | 20 (20) | 3 (15) | 4 (4) | 16 (20) | 20 (20) | 3 (15) | 4 (4) |
| DENV-4 | 1.05 x 10^8^ | 15 (15) | 8 (10) | 0 (1) | 6 (10) | 7 (15) | 7 (10) | 0 (1) | 4 (10) |
| **DENV-4 combined %** | | **100** | **93** | **19** | **71** | **66** | **90** | **19** | **57** |
