# Supplementary material for: Impact of temperature on patient-derived dengue virus breakthrough infections in wMel-infected Aedes aegypti
Source: PLoS Negl Trop Dis. 2026 Oct 9;20(10):e0014255. doi: 10.1371/journal.pntd.0014255 (PMC13686452; doi:10.1371/journal.pntd.0014255)
Supplement: Supplementary file 2 [file pntd.0014255.s002.docx]

**Supp. Table. 2. Adjusted marginal logistic regression models for the odds of viral infection in the head/thorax tissue.**

| **Head/thorax tissue** | **Adjusted odds ratio** | **95% confidence interval** | ***p* value** |
| --- | --- | --- | --- |
| (Intercept) | 1.06 | (0.07; 15.10) | 0.964 |
| WT (reference) |  |  |  |
| *w*Mel 28°C | 0.01 | (0.00; 0.03) | <0.001 |
| *w*Mel 31°C | 0.38 | (0.16; 0.89) | 0.027 |
| 28°C (reference) |  |  |  |
| 31°C WT | 0.39 | (0.12; 1.30) | 0.124 |
| 31°C *w*Mel | 16.75 | (8.51; 32.95) | <0.001 |
| DENV-1 (reference) |  |  |  |
| DENV-2 | 0.37 | (0.15; 0.87) | 0.023 |
| DENV-4 | 0.12 | (0.04; 0.33) | <0.001 |
| Interaction term: wMel*31°C | 43.28 | (10.79; 173.55) | <0.001 |
| log_10_ patient RNAemia | 2.00 | (1.49; 2.68) | <0.001 |
