# Supplementary material for: Impact of temperature on patient-derived dengue virus breakthrough infections in wMel-infected Aedes aegypti
Source: PLoS Negl Trop Dis. 2026 Oct 9;20(10):e0014255. doi: 10.1371/journal.pntd.0014255 (PMC13686452; doi:10.1371/journal.pntd.0014255)
Supplement: Supplementary file 3 [file pntd.0014255.s003.docx]

**Supp. Table. 3. Adjusted marginal logistic regression models for the odds of viral infection in saliva-inoculated mosquitoes.**

| **Mosquitoes inoculated with saliva** | **Adjusted odds ratio** | **95% confidence interval** | ***p* value** |
| --- | --- | --- | --- |
| (Intercept) | 4.35 | (0.82; 23.12) | 0.085 |
| WT (reference) |  |  |  |
| *w*Mel 28°C | 0.19 | (0.12; 0.31) | <0.001 |
| *w*Mel 31°C | 0.61 | (0.39; 0.96) | 0.032 |
| 28°C (reference) |  |  |  |
| 31°C WT | 1.90 | (1.23; 2.93) | 0.004 |
| 31°C *w*Mel | 6.13 | (3.69; 10.18) | <0.001 |
| DENV-1 (reference) |  |  |  |
| DENV-2 | 0.21 | (0.13; 0.34) | <0.001 |
| DENV-4 | 0.49 | (0.27; 0.90) | 0.021 |
| Interaction term: *w*Mel*31°C | 3.22 | (1.66; 6.26) | <0.001 |
| log_10_ patient RNAemia | 0.99 | (0.82; 1.19) | 0.907 |
